# Gut microbiota trigger host liver immune responses that affect drug-metabolising enzymes

**DOI:** 10.3389/fimmu.2024.1511229

**Published:** 2024-12-11

**Authors:** Jiaoyu Rao, Peng Qiu, Yonggang Zhang, Xiaokang Wang

**Affiliations:** ^1^ Department of Pharmacy, Shenzhen Longhua District Central Hospital, Shenzhen, China; ^2^ Clinical Laboratory Department, Shenzhen Longhua District Central Hospital, Shenzhen, China

**Keywords:** gut microbiota, liver immune response, drug-metabolising enzymes, precision medicine, epigenetic regulation

## Abstract

There is increasing evidence that the intestinal microbiota plays an integral role in disease pathogenesis and treatment. Specifically, the intestinal microbiota significantly influences the pharmacokinetics and pharmacodynamics of orally administered drugs through direct involvement in drug metabolism and, consequently, drug bioavailability. However, the gut microbiota also exerts immunoregulatory effects on the liver—the organ primarily responsible for drug metabolism—thereby indirectly impacting the body’s capacity to metabolise and process drugs. Individual differences in this pathway substantially contribute to the variability in clinical drug treatment outcomes observed between patients. This review examines the impact of liver immune responses, as triggered by the intestinal microbiota, on the activity of drug-metabolising enzymes and discusses the implications for precision medicine.

## Introduction

In regulating the host’s adaptation to the environment, the gut microbiota influences internal physiology and overall health. Indeed, essential roles for the gut microbiota in immune modulation, metabolic regulation, and organ development and morphogenesis have been demonstrated (1). This complex interplay between the host and its microbiota reflects an evolutionary dynamic in which both have adapted to achieve optimised coexistence. For instance, the gut microbiota provides essential nutrients and enhances immune responses, whereas host regulation ensures that the gut microbial community remains beneficial rather than harmful to health (2). Significant variations in gut microbiota composition among individuals reflect dietary, environmental, and genetic influences, which can result in differing health outcomes. These variations highlight the importance of understanding the microbiome’s role in human genetic adaptation and health (3). Interactions between the host and gut microbiota can give rise to emergent phenotypes, affecting both physiological traits and behaviours, which then influence host-environment interactions (4). In this context, the gut microbiota is a vital component of the host’s ecosystem, shaping physiological and behavioural adaptations in response to environmental challenges. It is therefore integral to the host’s adaptation, functioning as a dynamic ecosystem that influences health and development. An understanding of the interactions that underlie host-microbiota relationships will facilitate strategies for maintaining health and treating diseases (5).

The gut microbiome, often referred to as the “second genome,” substantially influences the efficacy and toxicity of a wide range of xenobiotics, including pharmaceuticals, dietary compounds, and environmental toxins (6). This intricate relationship between microbial inhabitants and therapeutic outcomes suggests a pivotal role for the gut microbiome in precision medicine strategies, whereby patients are treated based on their unique microbiota profiles. Moreover, the gut microbiota plays an essential role in host metabolism, particularly in energy homeostasis and glucose regulation (7). These findings underscore the importance of the microbiome in drug metabolism, as well as broader metabolic processes that affect health and disease states. The remarkable resilience of microbial species such as *Bacteroides thetaiotaomicron* during antibiotic treatments demonstrates the microbiome’s capacity to adapt and maintain gut health even under adverse conditions (8). The implications of these discoveries extend beyond gastrointestinal health, as evidenced by recent investigations into the genitourinary microbiome, which revealed its potential involvement in the pathogenesis of bladder, kidney, prostate, and possibly other cancers (9). This evidence indicates that the interplay between the microbiome and drug metabolism may also influence cancer treatment responses. Further research is required to fully elucidate the mechanistic roles of microbial communities in drug efficacy, cancer therapy, and overall health and disease.

Research concerning the impact of the gut microbiota on drug metabolism has primarily focused on two aspects that influence drug treatment outcomes: (i) the decomposition and metabolism of drugs by the gut microbiota, with subsequent effects on drug absorption and distribution; and (ii) the gut microbiota’s regulation of drug metabolism in the liver via the gut-liver axis—the principal route of communication between the gut microbiota and the liver—and its impact on liver function and immune responses (10). The liver is continually exposed to various metabolites and antigens derived from the gut microbiota and is therefore a central immunological organ. Studies have demonstrated that the composition of the gut microbiota modulates the immune environment of the liver, which then affects drug metabolism. Similarly, certain microbial metabolites have been shown to enhance or inhibit the activity of liver enzymes responsible for drug metabolism, thereby altering the pharmacokinetics of various medications (11). This interaction underscores the importance of understanding the gut microbiome’s role in liver immunology, particularly in the context of liver diseases where the gut-liver axis may be disrupted (12). Moreover, the liver’s capacity to process xenobiotics, including drugs, is influenced by signalling pathways activated by gut-derived metabolites. These metabolites can regulate the expression of genes involved in drug metabolism and detoxification, illustrating the intricate relationships among the gut microbiota, liver function, and drug metabolism (13). Elucidation of the mechanisms by which the gut microbiota affects liver immune responses is essential for efforts to develop targeted therapeutic strategies that consider the microbiome’s role in drug metabolism and liver health (8).

## Functions of immune cell populations in the liver

Immune cells play a central role in modulating acute and chronic liver diseases through inflammation and immunity. The liver, as a frontline immune organ, is uniquely positioned to detect and respond to pathogens entering the body via the gut. It contains the largest collection of phagocytic cells, which are essential for capturing and clearing bacteria, viruses, and other harmful substances (14). However, the liver is also exposed to numerous harmless foreign molecules, such as food antigens, resulting in a default state of immunotolerance. This balance between immunity and tolerance is crucial for maintaining liver function. Excessive inflammation can cause sterile hepatic injury and tissue damage, whereas an inadequate immune response can lead to chronic infections and cancer (14). The liver’s immune response is further complicated by interactions among its diverse cell populations. Recent studies have identified a proinflammatory hepatocyte subpopulation that plays a critical role in recruiting macrophages and suppressing T-cell responses during endotoxemia. This process involves complex signalling pathways, including CCL2-CCR2 and the PD-1/PD-L1 axis, which modulate immune activity in the liver (15).

Chronic liver diseases are characterised by persistent hepatocellular injury, which triggers a proinflammatory state capable of progressing to fibrosis, cirrhosis, and liver failure. The activation of inflammasomes—intracellular multiprotein complexes—is a key driver of this inflammatory response. Inflammasomes respond to cellular danger signals by activating caspase-1, which leads to the release of proinflammatory cytokines such as IL-1β and TNF-α. This cascade sustains hepatic inflammation and promotes the recruitment of adaptive immune cells, further exacerbating liver damage (16, 17).

The dynamic nature of liver inflammation, including interactions between immune cells and the liver parenchyma, can be visualised in real time using intravital imaging techniques. This capability to observe immune cells as they coordinate their activities in response to both acute and chronic liver diseases has revealed the complexity of the hepatic immune response (18). Research into these intricate immune mechanisms will accelerate the development of novel therapeutic strategies aimed at modulating liver inflammation and improving outcomes for patients with liver disease (**Table 1**).

**Table 1:** Gut microbiota trigger host liver immune responses by immune cells.

Disease	Functions	Reference
**Neutrophils**	Tissue repair and contribute to drug metabolism through the gut-liver axis	(20, 23)
**Monocytes and macrophages**	Express activation markers such as CD68 and further differentiate into an inflammatory M1 phenotype, an alternatively activated M2 phenotype, or an intermediate phenotype	(27–29)
**Dendritic cells**	Immune tolerance and act as key modulators of the immune response	(41, 42).
**NK and NKT cells**	Influence therapeutic outcomes in many diseases, through mechanisms that include cytotoxic granule release and pro-inflammatory cytokine production	(51)
**T cells**	CD8 T-cell recruitment to the liver is independent of antigen specificity and plays a critical role in viral hepatitis	(61)
**B cells**	Bacterial products trigger DCs to promote B-cell infiltration or activation in autoimmune liver diseases	(63)

The bold texts are names of the immune cells.

## Neutrophils

Neutrophils are the first responders to tissue injury and bacterial infections. The plasticity of these cells enables them to traverse endothelial barriers and migrate into the liver parenchyma (19). By engulfing bacteria and damaged cells, neutrophils play a key role in tissue repair and contribute to drug metabolism through the gut-liver axis (20). Although neutrophils historically have been viewed as agents of inflammation, recent studies have highlighted their essential functions in tissue repair and homeostasis. For example, neutrophils participate in the clearance of cellular debris and orchestrate the healing response. In addition to phagocytosing pathogens and dead cells, they release signalling molecules that recruit other immune cells to sites of injury, thereby enhancing the repair process (21). Neutrophils also actively contribute to the revascularisation of damaged tissues, indicating that their role extends beyond inflammation to include critical functions in tissue regeneration (22).

Bidirectional communication between the gut and liver is essential for maintaining homeostasis and responding to systemic challenges. By interacting with the gut microbiota, neutrophils influence the compositions of microbial communities, which then affect liver function and drug metabolism (23). Disruptions of the gut-liver axis, caused by factors such as alcohol or other stressors, can alter immune responses and contribute to the development of liver disease (23).

In summary, the emerging understanding of neutrophils’ roles in tissue repair, pathogen and damaged cell clearance, and drug metabolism through their interactions within the gut-liver axis highlights the need for further research into these multifaceted cells. This is particularly important in the context of chronic diseases, where tissue repair processes are often impaired (24).

## Monocytes and macrophages

As innate mononuclear phagocytes, monocytes and macrophages are highly sensitive to the tissue microenvironment (25). Circulating monocytes differentiate into macrophages upon reaching the liver, where they express activation markers such as CD68 and further differentiate into an inflammatory M1 phenotype, an alternatively activated M2 phenotype, or an intermediate phenotype (26). M1 macrophages are characterised by the expression of specific cell surface markers and elevated production of pro-inflammatory cytokines. M2 macrophages express mannose receptors, CD206, CD163, and arginase; they secrete anti-inflammatory cytokines such as IL-10 and TGF-β (27–29). Liver-resident Kupffer cells (KCs)—macrophages identified by F4/80 expression—play a pivotal role in many chronic liver diseases.

Monocytes and macrophages are key players in the immune response, involved in pathogen clearance and influencing therapeutic outcomes by modulating drug metabolism. Moreover, macrophages can alter metabolic pathways in response to various stimuli, affecting their capacity to process and respond to drugs. This metabolic flexibility enables macrophages to adapt to diverse environmental cues, including the presence of therapeutic agents, thereby impacting drug efficacy and resistance (30, 31). Numerous studies have emphasised the significance of macrophage polarisation in drug metabolism, as the balance between M1 (pro-inflammatory) and M2 (anti-inflammatory) phenotypes can profoundly influence how these cells interact with drugs. M1 macrophages are typically associated with heightened inflammatory responses and may enhance the clearance of certain drugs; M2 macrophages, which are involved in tissue repair and the resolution of inflammation, may promote drug resistance by fostering a more protective microenvironment (32, 33). Additionally, the metabolic reprogramming of macrophages during inflammation can result in the production of cytokines and enzymes that modulate drug metabolism. For example, the expression of cytochrome P450 (CYP450) enzymes—critical for drug metabolism—is influenced by the metabolic state of macrophages (34, 35). This interplay between macrophage metabolism and drug responses highlights the need for a deeper understanding of how these immune cells affect therapeutic strategies, particularly in chronic diseases where inflammation is a prominent factor (36, 37).

## Dendritic cells

The liver is unique in its capacity to induce systemic immune tolerance, which is vital for preventing excessive immune reactions to food antigens, commensal bacteria, and transplanted organs (38). Dendritic cells (DCs) are categorised into plasmacytoid and myeloid subtypes based on their origin, surface receptor expression, and function. Immature DCs predominantly reside in the liver, where they contribute to immune tolerance and act as key modulators of the immune response, particularly in the context of liver transplantation and chronic liver diseases. Recent studies have elucidated mechanisms through which immature DCs promote immune tolerance. For instance, they secrete anti-inflammatory cytokines such as IL-10, which suppresses T-cell activation and promotes the differentiation of regulatory T cells (Tregs), further enhancing tolerance (39, 40). Additionally, immature DCs interact with hepatic stellate cells and other liver-resident immune cells, establishing a microenvironment that favours immune regulation over activation (41, 42). The therapeutic potential of immature DCs has also been investigated. For example, genetically modified immature DCs expressing tolerogenic factors such as TGF-β1 and Fas ligand (FasL) have shown promise in enhancing immune tolerance in liver transplantation models (43). This approach aims to minimise the need for long-term immunosuppression, which is often associated with significant side effects. The ability of immature DCs to modulate immune responses also influences drug metabolism and clearance, thereby affecting drug efficacy and safety [1]. Studies of the interplay among immature DCs, liver immunology, and drug metabolism are crucial for developing strategies to optimise therapeutic interventions in liver-related pathological conditions. The liver is enriched with TGF-β, IL-10, and prostaglandins, which inhibit the maturation of DCs. However, in response to stimulation by the tissue microenvironment or pathogens, immature DCs mature and express surface markers and T-cell-stimulating mediators, such as IL-12 (44, 45). Mature DCs are the most effective antigen-presenting cells (APCs) for activating T cells. Several subsets of mature DCs have been identified. Plasmacytoid DCs (CD123^+^ BDCA1^+^) express high levels of TLR7 and TLR9, produce large amounts of IFN-α, and respond to viral pathogen-associated molecular patterns (46). Myeloid DCs are further classified into typical myeloid DCs (expressing CD11c, CD13, CD33, and CD11b), type I myeloid DCs (CD1c^+^), and type II myeloid DCs (CD141^+^ or BDCA3^+^) (47).

## Natural killer cells and natural killer T cells

NK and NKT cells are distinct from T and B cells in that they lack antigen receptors with somatic diversity (48). NK cells constitute 50% of human liver lymphocytes but represent only 5–20% of circulating lymphocytes. NKT cells express T-cell receptors (TCRs) along with NK cell markers from the C-type lectin superfamily, such as NK1.1. The major TCRs of NKT cells are invariant, including Val4/Ja281 in mice and Va24/JaQP in humans (49, 50). Classical invariant NKT cells recognise their CD1d-restricted ligand through mediator release induced by α-galactosylceramide. NK cells recruit NKT cells to the liver, where they utilise membrane-bound effector molecules, such as FasL and CD40, and secrete cytotoxic mediators, including granzyme B and perforin, from intracellular vesicles.

Both NK cells and NKT cells are integral to the immune response; their functionality can be modulated by metabolic pathways critical for activation and survival. Considering the central roles of these cells in drug metabolism and resistance pathways, they influence therapeutic outcomes in many diseases, particularly cancer. NK cells recognise and eliminate both tumour cells and virally infected cells through mechanisms that include cytotoxic granule release and pro-inflammatory cytokine production (51). Recent studies have highlighted the importance of NK cell metabolism in determining effector functions, suggesting that alterations in metabolic pathways can impair NK cell responses in chronic diseases (52). For instance, the mechanistic target of rapamycin (mTOR) complex 1 (mTORC1) has been identified as a key regulator of NK cell metabolism, promoting glycolytic pathways essential for cell activation and function.

Similarly, NKT cells, which share characteristics with both NK cells and conventional T cells, influence the tumour microenvironment and the efficacy of immunotherapies through their production of cytokines that either enhance or suppress immune responses (53). NKT cells also respond to lipid antigens and modulate the immune landscape, potentially affecting tumour responses to therapeutic agents and drug metabolism (54). Furthermore, the metabolic state of NK and NKT cells influences their ability to resist drug-induced apoptosis, contributing to therapeutic resistance (55). Insights into the metabolic regulation of these cells could lead to novel therapeutic strategies for enhancing their anti-tumour activity and overcoming resistance mechanisms. For example, targeting specific metabolic pathways could restore the functionality of exhausted NK and NKT cells in the tumour microenvironment, thereby improving the efficacy of existing cancer therapies. The interplay between NK and NKT cell metabolism and drug resistance pathways is a critical area of research that holds promise for the development of more effective immunotherapeutic strategies against cancer and other diseases.

## T cells

T cells, identified by CD3 expression, are further categorised based on their expression of CD4 and CD8 and into α/β and γ/δ subtypes based on their T-cell receptors (TCRs); γ/δ T cells are predominantly expressed in the liver. The liver’s unique immune microenvironment promotes both local and systemic immune tolerance, a process involving CD4 T cells (56). The interaction between CD4 T cells and APCs dictates T cell differentiation into Th1, Th2, Treg, or Th17 subsets. Th1 CD4 T cells secrete IFN-γ and TNF-α; Th2 CD4 T cells secrete IL-4, IL-10, and IL-13; and CD4 Treg cells produce IL-10 and TGF-β. Th2 cells, which activate B cells and stimulate antibody production, are predominantly associated with autoimmune liver diseases (57). Th17 cells are regulated by cytokines such as IL-17 and IL-22 (58, 59). The opposing functions of Th17 and Treg cells maintain immune stability, whereas an imbalance can result in persistent inflammation and autoimmune disorders (60). The differentiation of both Th17 and Treg cells depends on TGF-β, but their response thresholds differ. Evidence suggests that Treg cell differentiation is linked to Th17 cell differentiation, depending on the cytokine environment. The activation of Th17 cells is associated with liver diseases caused by alcohol, viral infections, or autoimmune processes.

However, the majority of T cells in the liver express CD8 (61). Acting as cytotoxic lymphocytes, they induce apoptosis through FasL, secrete pro-inflammatory cytokines, and lyse target cells. CD8 T-cell recruitment to the liver is independent of antigen specificity and plays a critical role in viral hepatitis.

## B cells

B cells constitute a small portion of the hepatic lymphocyte population but are implicated in the development of primary biliary cholangitis (previously called primary biliary cirrhosis) and primary sclerosing cholangitis. The infiltration of B cells in the liver is greater in patients with primary biliary cholangitis than in patients with primary sclerosing cholangitis. In primary biliary cholangitis, the proportion of CD19^+^CD69^+^ B cells in the liver is significantly higher than in peripheral blood (62). The mechanism of B-cell homing to the liver is not fully understood, although it involves both CXCL13 and CXCR5. CXCL13 is primarily produced by myeloid DCs in the liver (63), but its secretion is also stimulated by human monocyte-derived DCs in response to lipopolysaccharide (LPS). These findings suggest that bacterial products trigger DCs to promote B-cell infiltration or activation in autoimmune liver diseases. A study of hepatitis C virus (HCV)-infected livers demonstrated a critical role for CXCL13 in B-cell infiltration and recruitment within the liver (64). Additionally, peripheral blood B cells can serve as hosts for HCV, contributing to viral persistence. Another study showed that B-cell depletion can inhibit liver fibrosis (65). Reducing B-cell number and function may therefore offer therapeutic potential for patients with liver fibrosis.

## The gut microbiota and liver inflammatory diseases

Among the bacterial species that constitute the human intestinal microbiota, those belonging to the phyla *Firmicutes*, *Bacteroidetes*, *Actinobacteria*, and *Proteobacteria* predominate. The diversity and abundance of the gut microbiota contribute to overall stability and normal gut function, maintaining an ecological balance (66). Increasing evidence suggests that the gut microbiota influences the onset, development, and progression of multiple liver-disease-related complications. For example, intestinal dysbiosis is closely associated with non-alcoholic fatty liver disease (NAFLD), including non-alcoholic steatohepatitis (NASH) and cirrhosis, severe alcoholic hepatitis, and primary sclerosing cholangitis. Bidirectional communication between the liver and small intestine via the gut-liver axis involves the biliary tract, portal vein, and systemic circulation; bile acids and intestinal metabolites serve as mediators. Intestinal metabolites are transported through the portal vein to the liver, altering its microenvironment and function. The liver filters nutrients, bacteria, toxins, and metabolites, then removes them via the biliary system.

**Figure 1:**
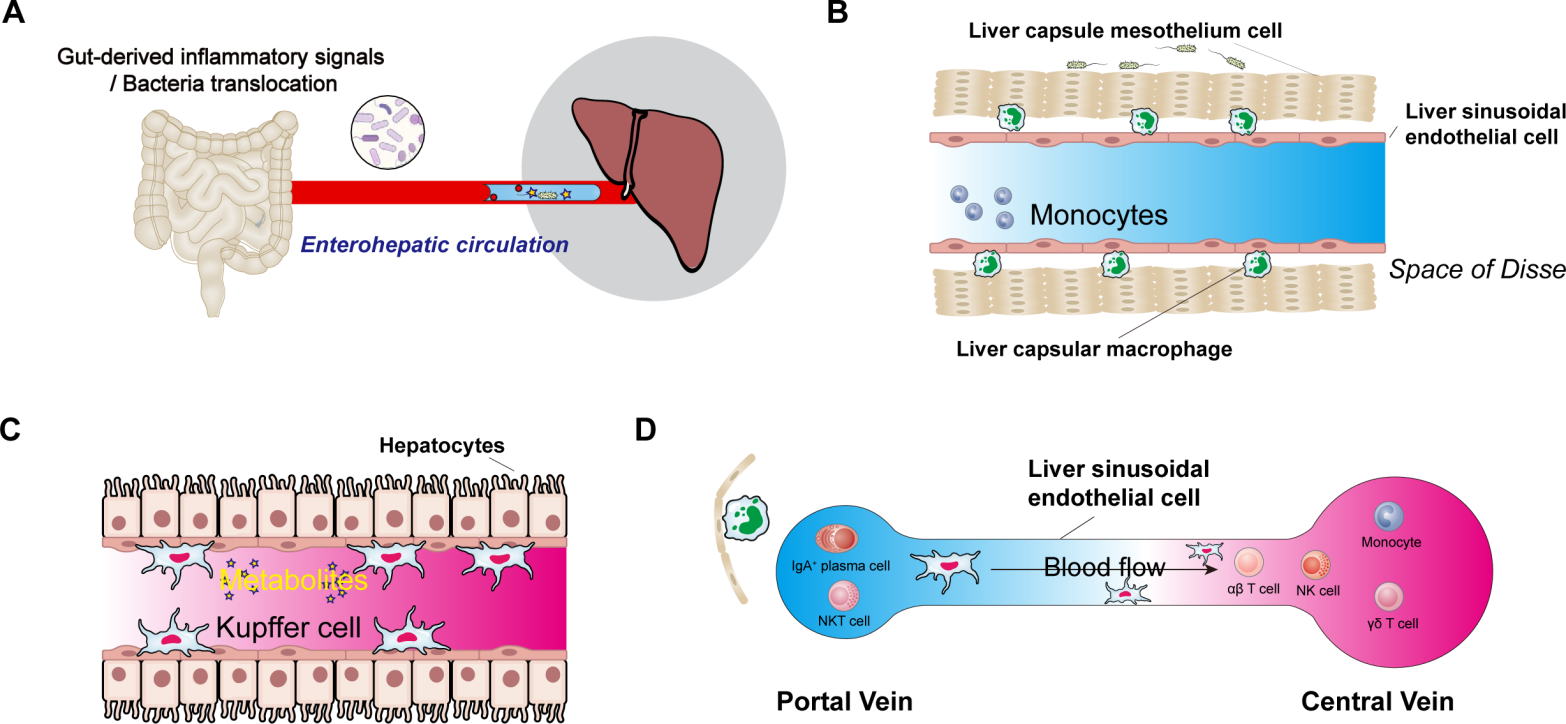
The central role of the intestinal microbiota and its metabolites in the liver’s immune system. **(A)**, Schematic diagram of the enterohepatic circulation. **(B)**, Immune cells in the space of Disse follow the circulation of gut microbiota and their metabolites via the gut-liver axis; liver sinusoidal endothelial cells, liver capsule mesothelial cells, and liver capsular macrophages are also present in the space of Disse. **(C)**, Commensal-derived metabolites, hepatic macrophages (Kupffer cells), and their potential effects on hepatocytes. **(D)**, Immune cells from the hepatic artery and portal vein converge in the liver sinusoids before draining into the central vein.

The gut-liver axis strongly modulates the liver’s immune response through the intestinal microbiota and its metabolites (**Figure 1A**). The liver constitutes approximately 10% of the body’s immune cells, and macrophages comprise 70% of the immune cell population (67). Liver lobules exhibit spatially polarised immune partitions, with high abundances of KCs, invariant NK (iNKT) cells, CD8^+^ tissue-resident memory (TRM) cells, and IgA^+^ plasma cells concentrated around the portal area. Liver capsule macrophages (LCMs), a distinct group of resident macrophages, are located in the liver capsule (**Figures 1B, C**). Each hepatic lobule consists of hepatocytes arranged around a central vein connected to the portal vein. The gradient between the portal vein and the central vein establishes a spatial division of labour among hepatocytes (**Figure 1D**). Immune cells, including NK cells, γδ T cells, CD4^+^ and CD8^+^ αβ T cells, monocytes, B cells, iNKT cells, mucosal-associated invariant T (MAIT) cells, and DCs, either circulate or temporarily patrol the liver sinusoids or parenchyma. Additionally, long-lived resident cells (e.g., KCs) are present. KCs are located exclusively within liver sinusoids and constitute 90% of liver sinusoidal wall macrophages. They are predominantly found in the midlobular and centrilobular regions, where they maintain close contact with sinusoidal endothelial cells; they also form connections with hepatic stellate cells and hepatocytes in the space of Disse. Their functions include engulfing and clearing circulating particles. The liver capsule, which contains portal zone and capsular macrophages, delineates the liver parenchyma from the peritoneal cavity (68). Portal zone LCMs develop from postnatal adult haematopoietic stem cell-derived monocytes and establish a cellular network within the liver’s protective capsule.

## The gut microbiota and toxic liver injury

The liver continuously processes small foreign molecules entering the portal circulation. As the starting point for sensing and biologically processing small molecules in the intestine, the liver acts as the first gate of metabolism. The microbiota responds to xenobiotics by altering microbial gene expression, generating small metabolites that affect the liver, making it a frequent site of poisoning. The Nrf2/ARE pathway protects the liver by activating drug-metabolising enzymes and transporters (69); Nrf2 activation protects against ischaemic liver injury (70). Mice lacking Nrf2 or the ability to biochemically activate this pathway are hypersensitive to exogenous insults, such as hepatotoxic drugs, due to the failure to upregulate canonical effector genes. Saeedi et al. observed bacterial activation of hepatic Nrf2 via the metabolite 5-methoxyindoleacetic acid, which protected the liver against the harmful effects of acetaminophen or ethanol (71). These findings suggest that alterations in the microbiota contribute to specific responses to potentially hepatotoxic drugs.

## The gut microbiota and alcoholic liver disease

Alcohol consumption can disrupt the gut microbiota before the onset of liver fibrosis symptoms. Metagenomic studies have revealed significant alterations in gut microbial diversity in response to heavy alcohol consumption, including an increase in *Proteobacteria* and a decrease in *Firmicutes* and *Bacteroidetes*. In patients with severe alcoholic liver disease (ALD) and alcoholic hepatitis, the intestinal abundance of *Bifidobacteria* and *Streptococci* is elevated, whereas that of *Lactobacilli* is reduced. In patients with chronic alcohol abuse, pathogenic *Candida* species proliferate in the intestine and enter the systemic circulation. Leaky gut, characterised by increased intestinal permeability, is a common condition in ALD patients. Dysbiosis reflects the alcohol-mediated degradation of natural defence proteins such as Reg3a, which increases microbial interactions with intestinal epithelial cells. Triggered by the resulting intestinal inflammation, monocytes and macrophages accumulate in the lamina propria and release TNF-α, impairing barrier function and thereby increasing intestinal permeability. This compromised gut barrier allows antigens, metabolites, and microorganisms to reach the liver via the portal circulation.

## The gut microbiota and NAFLD/NASH

The liver’s exposure to pathogens and gut microbial metabolites via the gut-liver axis creates a hepatic environment characterised by pro-inflammatory cytokines such as IL-1, IL-6, and TNF-α. In chronic pro-inflammatory conditions associated with alcohol use, drug intake, obesity, and diabetes, the generation of reactive oxygen species promotes liver damage and fibrosis, contributing to the onset and progression of ALD, alcoholic hepatitis, NAFLD, NASH, drug-induced liver toxicity, liver fibrosis, cirrhosis, and related complications. The interaction between the intestine and liver is a critical determinant of NAFLD. Moreover, NAFLD has been implicated in the development of obesity due to alterations in the gut microbiota that enhance nutrient absorption (72). Specifically, gut dysbiosis induced by lifestyle factors (e.g., a high-fat or low-fibre diet, physical inactivity, smoking, and alcohol consumption) contributes to the development of NAFLD and other liver diseases by weakening the intestinal barrier and promoting the translocation of bacteria or bacterial products into the portal circulation. A high-fat, low-fibre diet, irregular eating habits, a sedentary lifestyle, and antibiotic use contribute to the onset of metabolic syndrome, including obesity and diabetes, by disrupting the balance between alpha-diversity and intestinal ecology.

Dietary changes directly influence the intestinal microbiota, metabolic patterns, and metabolite composition, potentially compromising the intestinal vascular barrier. This disruption allows the transport of metabolites, toxins, chemokines, or cytokines to the liver via the portal circulation. In a study of mice fed a high-fat diet, intestinal vascular barrier dysfunction caused by dysbiosis led to increased LPS absorption and elevated serum LPS-binding protein levels. This was accompanied by heightened expression of TLR4 and TNF-α in hepatocytes, resulting in liver inflammation. Irregular intestinal barriers, leaky gut symptoms, elevated plasma LPS levels, and increased TLR4 expression are hallmarks of NASH.

Microbial penetration of the liver capsule is hindered by the protective effect of LCMs. Japanese researchers identified a specific bacterial family, *Odoribacteraceae*, in the portal vein area of the liver near the intestine. These bacteria contribute to the formation of an immunosuppressive microenvironment by synthesising isohelolithocholic acid, which induces the production of Marco^+^ immunosuppressive macrophages. These macrophages express high levels of IL-10 and the scavenger receptor Marco, which sequesters pro-inflammatory pathogen- and damage-associated molecular patterns (PAMPs and DAMPs, respectively), thereby limiting excessive inflammation at the liver entrance. A leaky intestinal barrier exacerbates inflammation, particularly in the portal vein area. This effect is intensified when Marco^+^ macrophages are significantly reduced, as occurs in primary sclerosing cholangitis, NASH, and other chronic liver inflammatory diseases (73).

Alterations in metabolic pathways, including increased ethanol production, reduced short-chain fatty acid (SCFA) synthesis, and disruptions in choline metabolism and bile acid balance, are associated with the development of NAFLD. In obese children with NASH, elevated blood ethanol concentrations result from an overgrowth of *Enterococcus faecium* B6 in the dysbiotic gut. Endogenous ethanol absorbed into the bloodstream is transported via the portal vein to the liver, where it exacerbates oxidative stress. Research in humans and animals has demonstrated a link between reduced SCFA levels and the onset of metabolic syndrome and NAFLD. Additionally, increased growth of *Proteobacteria* and a decrease in *Bacteroidetes* contribute to the dietary fibre maldigestion observed in NAFLD. SCFAs play a protective role against NAFLD; acetate administration reverses steatosis, while enhancing hepatic mitochondrial activity and overall liver function. SCFAs, particularly propionate, also down-regulate the expression of gluconeogenic enzymes in hepatic tissue. Moreover, SCFAs inhibit insulin signalling in adipose tissue by activating G-protein-coupled receptor 43 (GPR43), which limits lipid accumulation in adipocytes (74). Empirical evidence suggests that reduced SCFA levels are associated with increased hepatic lipid accumulation and disruption of the intestinal barrier, thereby promoting NAFLD.

Butyrate, an energy substrate, mitigates intestinal inflammation and modulates satiety. It also plays a critical role in maintaining intestinal homeostasis by enhancing intestinal barrier integrity, preventing the translocation of toxins or antigens.

## The gut microbiota and MAFLD

Microbiota-derived secondary bile acids have been implicated in glucose metabolism and obesity, and consequently in metabolic dysfunction–associated steatotic liver disease (MAFLD), where dysbiosis is also a prominent feature. Guanosine diphosphate (GDP) entering the liver via the portal circulation may trigger an inflammatory response through TLR and interferon signalling, as well as by activating macrophages and other inflammatory cell subsets (75), suggesting a close link between GDP and immunity.

As MAFLD progresses to metabolic steatohepatitis, there is an increase in all inflammatory cell types, including macrophages, lymphocytes, and granulocytes (76). Factors contributing to this progression include gut microbiome signals that reach the liver via the portal vein due to altered intestinal permeability and damaged fatty liver cells. Increases in immune and inflammatory cells are correlated with aggravated liver injury and MAFLD progression, whereas an increase in anti-inflammatory cells is associated with disease regression. IL-10 has been shown to suppress immune responses by modulating both innate and adaptive systems. It also plays a critical role in preventing liver inflammation caused by commensal bacteria in periportal macrophages, suggesting a role in disease prevention. In a paediatric study (trial number NCT02842567), treatment with hydroxytyrosol and vitamin E alleviated NAFLD-associated systemic inflammation by increasing circulating IL-10 levels (77).

In humans, conventional DCs are categorised into type 1 (cDC1) and type 2 (cDC2). The proliferation of cDC1 may exacerbate liver inflammation by activating CD8^+^ T cells. TNF secretion by monocytes and macrophages is induced by the microbiota shortly after birth. Microbiota-associated myeloid TNF enhances the ability of pro-cDC1 to elicit protective CD8^+^ T-cell responses by regulating their secretion of IL-10 and IL-12 p40 (78, 79). In patients and mice with MAFLD and associated dysbiosis, faecal microbiota transplantation has been shown to increase the levels of beneficial bacteria, reduce the abundance of pathogenic bacteria, and ameliorate hepatic steatosis.

The downregulation of group 3 helper innate lymphoid cells (ILC3s) by the liver-homing chemokine receptor CXCR6 results in an abnormal ILC3 distribution in MAFLD patients and mice. This distribution is characterised by a significant decrease in ILC3s in the liver and an increase in ILC3s outside the liver. Furthermore, an inverse correlation has been reported between disease severity and the proportion of hepatic ILC3s, with a corresponding reduction in hepatic steatosis (80).

## The gut microbiota and cirrhosis

Cirrhosis represents the final stage of liver damage caused by various factors, including ALD, NAFLD, NASH, or infection. Its pathological features include hepatocellular decline, the progression of fibrosis and regenerative nodules, and impaired liver function. As in other liver diseases, cirrhosis is characterised by the translocation of bacteria or their metabolites to the liver, resulting from a deterioration of intestinal barrier function or a leaky gut. The transport of *Escherichia coli* capsular LPS into the liver and the induction of TLR4-mediated signalling pathways have been observed in cirrhosis. TLR4 is expressed on parenchymal and non-parenchymal liver cells; it functions as both a PAMP and a DAMP. A substantial number of haematopoietic stem cells have also been observed in the intervertebral disc space in cirrhosis. The interaction of haematopoietic stem cell TLRs and co-receptors with LPS triggers signalling cascades that activate pro-inflammatory cytokines (IL-6, IL-8, TNF-α), chemokines (MCP-1, MIP-2, ICAM-1), and the release of the anti-apoptotic protein Bcl-2. The chemokines and cytokines released by activated haematopoietic stem cells stimulate leukocyte infiltration, leading to hepatocyte destruction and further aggravation of the fibrotic response by activating quiescent haematopoietic stem cells in a vicious cycle. Metagenomic studies of the gut microbiota have demonstrated that reductions in alpha-diversity and changes in beta-diversity are frequently associated with cirrhosis. These changes include a dominance of *Enterobacteriaceae*, *Enterococcus*, and *Staphylococcus*, along with reductions in *Ruminococcaceae* and *Lachnospiraceae*. Consequently, the gut microbiota can promote liver cirrhosis by increasing the abundance of LPS-prone species (81).

## Effect of the hepatic immune inflammatory response on drug metabolism

Liver disease is accompanied by extensive changes in the body’s distribution, metabolism, excretion, and toxicity (DMET) pathways (**Table 2**). Alcohol-induced intestinal dysbiosis in patients with ALD disrupts bile acid homeostasis, exposing the liver to toxic bile acids. Bile acid deconjugation interferes with FXR activation in enterocytes, reducing plasma levels of FGF-15 and increasing the activity of CYP7A1 in hepatocytes. Because alcohol is detoxified by alcohol dehydrogenase, the development of ALD was initially attributed to malnutrition resulting from the hepatic metabolism of alcohol. However, the discovery of the microsomal ethanol oxidation system (MEOS) changed this perspective. In MEOS, CYP2E1 is one of the primary ROS generators in the liver and is considered a key factor in ALD. Recent studies have shown that human CYP2A6 and its mouse analogue CYP2A5 are also induced by alcohol; the mouse analogue is dependent on CYP2E1. Unlike CYP2E1, CYP2A5 appears to prevent the occurrence of ALD.

**Table 2:** Dysregulation of drug-metabolising enzymes and transports in liver disease.

Disease	Target Genes	Expression Change	Reference
Obstructive cholestasis	MRP3	MRP3	(82)
Chronic hepatitis	CYP1A2, CYP2E1, CYP3A4, NTCP, OATP1B1, OCT1	↓CYP1A2, CYP2E1, CYP3A4, NTCP, OATP1B1, OCT1	(83)
HBV-positive hepatocellular carcinoma	CYP1A2, CYP3A4	↓CYP1A2, CYP3A4, CYP2C, CYP2D6, CYP2A6, CYP2E1, CYP1A2, CYP3A4	(87)
MAFLD	CYP1A2, CYP2C9, CYP2C19, CYP2D6, CYP3A4	CYP1A2, CYP2C9, CYP2C19, CYP2D6, CYP3A4	(88)
ALD	CYP2A6	↑ CYP2A6	(89)
IBD	CYP1A2, CYP2E1, CYP2A5	↓CYP1A2, CYP2E1, CYP2A5	(90, 91)
Crohn’s disease	CYP3A4, P-gp	↓CYP3A4, P-gp	(92)
NASH	CYP2B1, CYP2E1, CYP2C19, UGT1A9, UGT2B10, SULT1C4, UGT3A1, SULT1A1, SULT4A1	↑ CYP2B1, CYP2E1 ↓CYP2C19 ↑ UGT1A9, UGT2B10, SULT1C4, UGT3A1, SULT1A1, SULT4A1	(85, 93)

↑ increased expression, ↓ decreased expression; CYP, cytochrome P450 enzyme. Yellow represents upregulated gene expression; Blue represents down-regulated gene expression; Black represents changes in gene expression.

In a study of patients with cholestatic liver disease, Chai et al. found that TNF-mediated activation of the c-Jun N-terminal kinase (JNK)/stress-activated protein kinase (SAPK) pathway led to a substantial increase in liver disease symptoms. Both the mRNA and protein levels of the cell membrane protein MRP3 were significantly higher in patients with cholestasis than in those without, by 3.4- and 4.6-fold, respectively (82). Nakai et al. observed significantly lower expression of the genes encoding CYP1A2, CYP2E1, CYP3A4, OATP1B1, and OCT1 in patients with chronic hepatitis C cirrhosis relative to healthy controls (83). A meta-analysis of 16 independent studies revealed a significant reduction in CYP2C19 expression to 46% of control levels in NASH patients, with a further decrease to 43% in those with severe fibrosis (84). Cho et al. demonstrated that changes in liver metabolic enzymes caused by NASH affect drug metabolism. In rat models of NASH, the levels of CYP2B1 gene and protein expression in the liver were markedly lower than in healthy control rats. This inhibition of metabolic activity resulted in increased plasma levels of bupropion and decreased levels of its metabolite, hydroxybupropion, indicating significantly reduced bupropion clearance (85). Hardwick et al. examined changes in phase II metabolic enzymes during the progression of NAFLD. In patients with steatosis, fatty liver NASH, or cirrhotic NASH, the expression levels and activity of uridine diphosphate glucuronosyltransferases (UGTs) and sulphate transferases (SULTs) gradually increased with disease progression (86). The levels of UGT1A9, UGT2B10, and SULT1C4 expression were not significantly different between patients with steatosis and healthy controls, but they were significantly upregulated in NASH patients. Similarly, UGT3A1 gene expression was significantly higher in NASH patients than in those with steatosis. The expression of SULT1A1 and SULT4A1 was significantly higher in NASH patients with cirrhosis relative to such patients with steatosis; they were also significantly elevated in NASH patients with cirrhosis relative to healthy controls, patients with steatosis alone, and patients with fatty liver NASH. SULT1A1 activity is significantly increased in patients with steatosis but decreased in those with NASH, leading to disruptions in the sulfonation of acetaminophen during NAFLD progression.

## Mechanism of action in the activity of drug-metabolising enzymes

**Figure 2:**
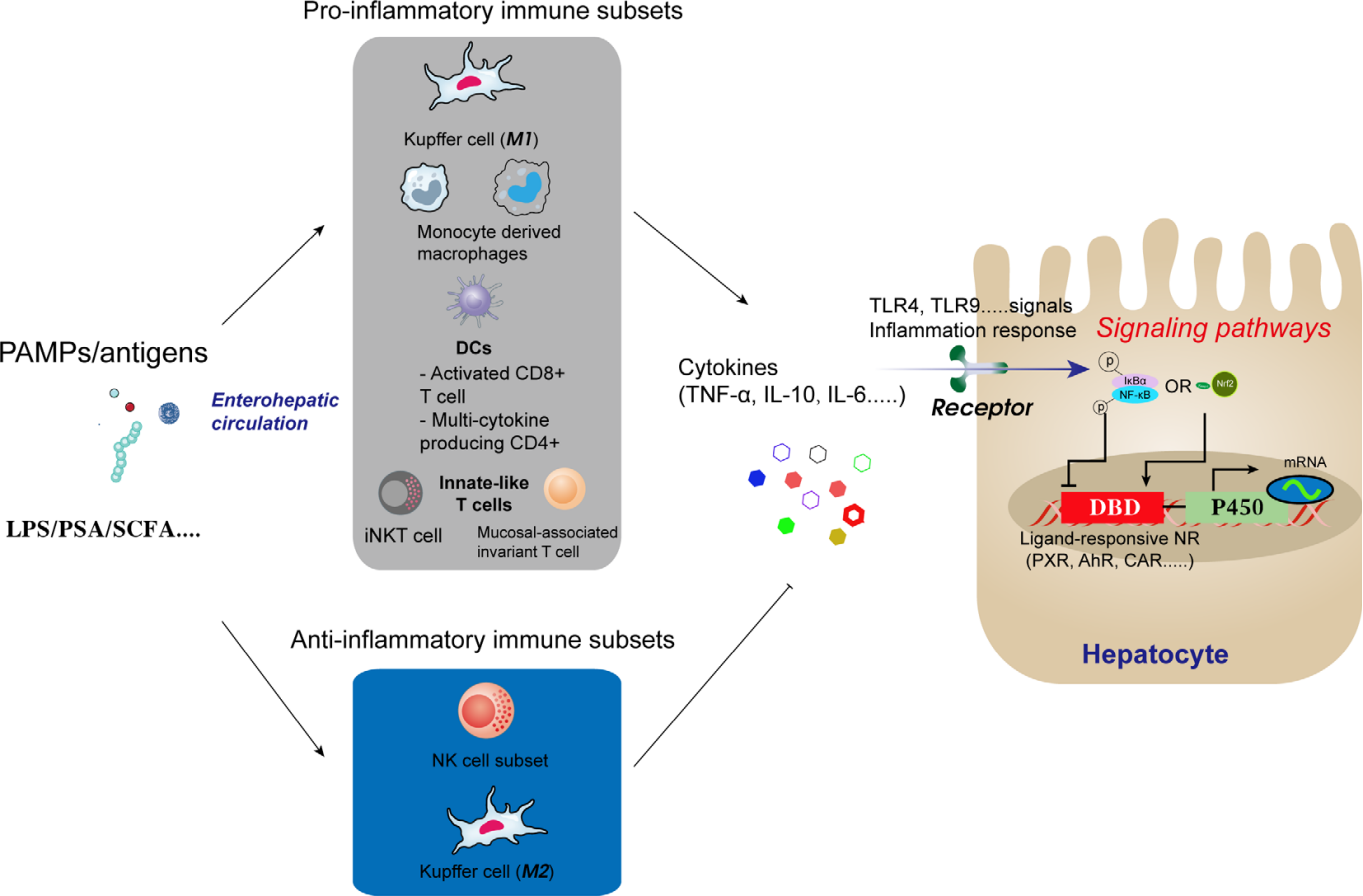
Antigens and other compounds circulate via the enterohepatic axis to stimulate macrophages and capsule macrophages in the hepatic portal region. Macrophages then trigger pro-inflammatory or anti-inflammatory immune responses depending on the stimulus.

The gut microbiota and its metabolites, such as LPS, polysaccharide A, and SCFA, are among the regulators of the liver’s immune response via the enterohepatic circulation. As illustrated in **Figure 2**, these substances or antigenic compounds traverse the gut-liver axis, stimulating macrophages and LCMs within the hepatic portal region, which then initiate either pro-inflammatory or anti-inflammatory immune responses.

The pro-inflammatory and anti-inflammatory activities of Kupffer cells (KCs) depend on their polarisation and include the promotion of TNF-α and IL-10 secretion. These cytokines indirectly influence NF-κB signalling in hepatocytes and regulate P450 gene transcription. Research by our group demonstrated that *Astragalus* polysaccharide, a traditional Chinese medicine, enhances hepatic voriconazole metabolism by counteracting the inhibition of CYP2C19 by NF-κB signalling in hepatocytes via the gut-liver axis (28). This finding further supports the capacity of the gut microbiota to positively or negatively influence the expression and activity of drug-metabolising enzymes.

## Regulation of metabolism by cytokine-nuclear receptors

Inflammatory cytokines participate in the regulation of DMET pathways (29, 94, 95). Mimura et al. reported that IL-6 suppresses CYP3A4 expression and enzymatic activity in human liver cancer cells. This suppression enhanced the efficacy of the chemotherapeutic agents gefitinib and paclitaxel in patients (96). Park et al. conducted cellular, animal, and clinical studies to investigate the regulation of ABC transporters by IL-8 in liver cancer. Their results indicated that the substantially elevated levels of IL-8 in liver cancer induce the expression of efflux transporters, thereby diminishing drug sensitivity (97). In some cases, cytokines exert dual regulatory effects on DMET pathways, depending on timing and dose. De Oliveira et al. found that in mice administered varying doses of LPS, a low dose (0.025–2.0 mg/kg) inhibited the activities of CYP2A5, CYP1A, and CYP2B, whereas a high dose (2 mg/kg) increased NO levels, reversing the downregulation of CYP2A5 without affecting the inhibition of CYP1A and CYP2B (98). Subsequent studies demonstrated that inflammation-related signalling pathways and nuclear receptors significantly influence cytokine-mediated regulation of DMET; the NF-κB pathway serves as the primary pathway involved in regulating metabolic enzyme transporters (99). Keller et al. reported that IL-6 suppresses retinoic acid X receptor α (RXRα) and the nuclear receptor constitutive androstane receptor (CAR) by activating MAPK and NF-κB signalling pathways. Their findings indicated that transcriptional repression of various drug-metabolising enzymes and transporters was induced through the modulation of farnesoid X receptor (FXR), liver X receptor (LXR), peroxisome proliferator-activated receptor (PPAR), pregnane X receptor (PXR), and vitamin D receptor (VDR) (100). Kusunoki et al. [35] demonstrated that in mice with colitis, LPS from the colon’s inflammatory site triggered a liver inflammatory response, enhancing NF-κB nuclear translocation while suppressing PXR and CAR mRNA expression and nuclear translocation. This reduction in hepatic CYP expression and activity resulted in elevated plasma drug concentrations and a higher incidence of adverse reactions (101). Nathan et al. performed *in vivo* and *in vitro* studies, revealing significant increases in IL-18 levels under cholestatic conditions. These levels activated the NF-κB signalling pathway, inhibiting FXR expression and subsequently altering MRP2 activity. The depletion of IL-18 normalised MRP2 levels.

## Epigenetic regulation of genes encoding drug-metabolising enzymes

The considerable interindividual variability in CYP450 enzyme expression impacts clinical pharmacotherapy. Numerous studies have demonstrated that genetic polymorphisms influence the expression of certain CYPs and, consequently, drug metabolism. However, their contribution to the observed individual differences is not fully understood. Epigenetic regulatory mechanisms appear to play a crucial role in explaining these differences in CYP expression (102). Epigenetic regulation refers to genome modifications that do not involve changes in the DNA sequence, such as DNA methylation, histone modification, and non-coding RNA regulation (103). These mechanisms are involved in modulating the activities of drug-metabolising enzymes in liver disease. The following discussion focuses on the epigenetic regulation of CYP2 and CYP3, drug-metabolising enzymes enriched in the liver.

The CYP2 family comprises 16 isoforms, including CYP2A6, CYP2B6, CYP2C (CYP2C8, CYP2C9, CYP2C19), CYP2D6, and CYP2E1. CYP2A6 is primarily expressed in the liver, where it constitutes 4% of total CYP expression (28). Its expression responds to PXR and CAR agonists, such as rifampicin and phenobarbital, as well as the glucocorticoid receptor (GR) agonist dexamethasone, with strong sex-based differences (104, 105). CYP2A6 is the main enzyme involved in metabolising nicotine, the anticancer drug 5-fluorouracil, and various prescription drugs (106). CYP2A6 mRNA and protein expression, along with CYP2A6 enzyme activity, vary more than 50-fold among individuals (107). This variability is attributed to genetic polymorphisms and epigenetic changes, including DNA methylation and histone modifications. In primary hepatocytes with high CYP2A6 activity, all CpG sites at the PXR/PGC-1α binding site of the CYP2A6 promoter region are demethylated; in hepatocytes with low activity, these sites are hypermethylated. This finding suggests that DNA demethylation regulates CYP2A6 expression via transcription factors such as PXR. The induction of CYP2A6 expression by dexamethasone has been shown to depend on histone H4 acetylation. Specifically, increased H4 acetylation in the proximal promoter region loosens chromatin structure, facilitating the binding of the nuclear transcription factor hepatocyte nuclear factor 4α (HNF4α) and thereby upregulating CYP2A6 transcription (105).

Research concerning epigenetic regulation of CYP3 (CYP3A4/A5/A7) has demonstrated that CYP3A4 mRNA expression in the human liver is significantly associated with H3K4me3 and H3K27me3 modifications at the promoter region of the CYP3A4 gene (108). The histone deacetylase inhibitor trichostatin A increases CYP3A4 transcriptional activity, while protein arginine methyltransferase 1 (PRMT1) catalyses histone arginine methylation. A role for PXR in regulating CYP3A4 expression (by upregulating H4R3 acetylation at the promoter region) has also been reported (109). Considering that histone modifications are associated with drug-induced CYP3A4 expression (110), further investigation of H3K4me3/H3K27me3 modifications at the CYP3A4 promoter region and PXR-mediated H4R3 acetylation influenced by the intestinal microbiota and its metabolites will provide valuable insights into the regulatory mechanisms of CYP3 expression in liver disease.

## Epigenetic regulation of nuclear receptor genes

Epigenetic changes in nuclear receptor expression (e.g., phosphorylation, methylation, acetylation, and ubiquitination) can affect the expression and activity of drug-metabolising enzymes and transporters. For example, the nuclear receptor PXR, which regulates the expression of CYPs and ABC transporters, is a target of epigenetic modifications caused by exogenous substances (111). In colon cancer cells, PXR expression is downregulated by DNA methylation, resulting in reduced CYP3A4 expression. In cells treated with the DNA methyltransferase inhibitor 5’-Aza-dC, PXR methylation was significantly reduced, whereas the expression levels of both PXR and CYP3A4 were significantly upregulated (109). The transcriptional activity of PXR is mediated by PRMT1, which PXR recruits to the promoter region of the CYP3A4 gene. This recruitment leads to the methylation of arginine 3 (H4R3) on histone H4, thereby upregulating CYP3A4 transcription. Post-translational modifications, such as phosphorylation, also play a critical role in regulating PXR-mediated gene expression. Phosphorylated PXR recruits a transcription repressor protein complex to the regulatory region of the target gene, repressing its transcription (112). Inhibition of the inflammatory pathway regulated by PXR has been shown to result from SUMOylation. SUMOylated PXR prevents the dissociation of the transcription repressor complex by binding to genes encoding pro-inflammatory factors responsive to NF-κB, thus inhibiting their expression (113). SUMOylation and ubiquitination control the stability, activity, and transcriptional repression of PXR through a regulatory circuit in hepatocytes. Both pathways are activated in a ligand- and TNFα-dependent manner. SUMOylation inhibits ubiquitination-induced degradation of PXR, increasing its stability (114). Additionally, SUMOylation suppresses PXR-mediated rifampicin-induced expression of CYP3A4 and ABCB1 (115). Therefore, changes in the post-translational modification status of PXR, such as SUMOylation, influence drug metabolism and result in altered drug phenotypes.

The hepatic nuclear receptor CAR regulates the body’s defences against damage from exogenous and endogenous toxic substances. Ligand-activated CAR translocates to the nucleus, where it induces the transcription of target genes, including those encoding drug oxidases and transporters (116). CAR expression is regulated by epigenetic modifications such as DNA methylation. A study of the CAR gene in HBV-induced liver cancer revealed high levels of methylation, leading to reduced CAR expression and consequently lower expression of the CYP2C19 gene (117). In HepG2 cells, the expression of CAR and its target genes (CYP2B6 and CYP3A4) is inhibited by berberine, which increases DNA methylation and interferes with CAR binding to gene promoters (118). Similarly, phenobarbital-induced changes in CYP2B10 gene expression in hepatocytes have been linked to CAR-mediated DNA methylation (119, 120). Accordingly, decreased DNA methylation of CAR may enhance the expression of target genes, mitigating the toxic effects caused by substrate accumulation.

## Epigenetic regulation of other nuclear receptors

The nuclear receptors subject to epigenetic regulation include HNF4α, a key transcription factor involved in metabolic processes, particularly in the liver and intestine. Cofactors such as transacetylase inhibitors (TSAs) and CCAAT/enhancer-binding protein alpha (C/EBPα) interact with HNF4α to fine-tune the expression of genes involved in lipid and glucose metabolism, as well as xenobiotic metabolism (121, 122). HNF4α also functions as a cofactor, together with PXR and CAR, in regulating the expression of genes encoding metabolic enzymes and those related to drug elimination in the liver (123). Protein expression levels of HNF4α and C/EBPα are upregulated by TSAs, maintaining the CYP-mediated phase I biotransformation capacity, whereas phase II glutathione S-transferase (GST) activity remains unaffected (124, 125). HNF4α cooperates with C/EBPα to regulate metabolic gene expression, particularly during liver development and in response to metabolic signals [6]. This cooperation is essential for maintaining metabolic homeostasis and adapting to nutritional changes.

An understanding of the epigenetic mechanisms governing HNF4α activity and its interactions with cofactors could provide insights into novel therapeutic strategies for metabolic diseases. However, the interplay between HNF4α and epigenetic modifications, such as histone acetylation and methylation, adds complexity to this regulatory landscape. For instance, TSAs have been shown to alter the acetylation status of histones and thereby influence the transcriptional activity of HNF4α and its target genes. In HepG2 cells treated with 5-azacytidine and vitamin C, the upregulation of HNF4α and E-cadherin induced an epigenetic transformation of the cells towards a primary human hepatocyte-like phenotype. This transformation included enhanced expression and activity of phase I metabolic genes and their encoded enzymes, including CYPs (126). The drug-induced expression of CYP1A1, CYP1A2, and CYP1B1 is regulated by the polycyclic aromatic hydrocarbon receptor. In HepG2 cells treated with 5-azacytidine and deoxycytidine, cytosine residues within CpG dinucleotides, including those in the xenobiotic response element, are partially demethylated. This demethylation restores RNA polymerase II and TATA-binding protein activity, thus promoting CYP1A1 expression.

In summary, the epigenetic regulation of nuclear receptors such as HNF4α, along with the involvement of cofactors such as TSAs and C/EBPα, is crucial for regulating metabolic processes. The development of epigenetic modifiers that enhance specific functions of drug-metabolising enzyme activity in hepatocytes will create new opportunities for improving drug metabolism testing using *in vitro* models. Further research in this field may reveal novel strategies for treating metabolic disorders and expand our understanding of the complex regulatory networks governing metabolism.

## Conclusion and opinions

Efforts to enhance treatment precision by considering the diversity of the gut microbiota have the potential to improve drug safety and efficacy. The liver is the primary organ involved in drug metabolism, but this function is strongly influenced by the gut microbial community. Increasing knowledge of the gut-liver axis has revealed that the gut microbiota is a promising target for innovative liver disease therapies. Preclinical studies have explored the use of phages and engineered bacteria to modify the gut-liver axis; however, these novel approaches have not been adequately validated in humans. Progress in this area has been hindered by the limitations of rodent models (even with human microbiota implantation), which fail to fully replicate the complexity of the human gut-liver axis. Nevertheless, if proven safe and effective in clinical trials, these therapies could revolutionise the management of liver inflammatory diseases and improve the stability and efficacy of clinical drug treatments. The epigenetic modulation of gene expression alters the expression and activity of drug-metabolising enzymes and nuclear receptors, contributing to interindividual variability in drug responses. A comprehensive investigation into the epigenetic regulation of drug metabolism will help to improve the safety and efficacy of clinical treatments.
